# Data-Driven Identification of Unusual Prescribing Behavior: Analysis and Use of an Interactive Data Tool Using 6 Months of Primary Care Data From 6500 Practices in England

**DOI:** 10.2196/44237

**Published:** 2023-04-19

**Authors:** Lisa EM Hopcroft, Jon Massey, Helen J Curtis, Brian Mackenna, Richard Croker, Andrew D Brown, Thomas O'Dwyer, Orla Macdonald, David Evans, Peter Inglesby, Sebastian CJ Bacon, Ben Goldacre, Alex J Walker

**Affiliations:** 1 Bennett Institute for Applied Data Science Nuffield Department of Primary Care Health Sciences University of Oxford Oxford United Kingdom; 2 Oxford Health NHS Foundation Trust Oxford United Kingdom

**Keywords:** dashboard, data science, EHR, electronic health records, general practice, outliers, prescribing, primary care

## Abstract

**Background:**

Approaches to addressing unwarranted variation in health care service delivery have traditionally relied on the prospective identification of activities and outcomes, based on a hypothesis, with subsequent reporting against defined measures. Practice-level prescribing data in England are made publicly available by the National Health Service (NHS) Business Services Authority for all general practices. There is an opportunity to adopt a more data-driven approach to capture variability and identify outliers by applying hypothesis-free, data-driven algorithms to national data sets.

**Objective:**

This study aimed to develop and apply a hypothesis-free algorithm to identify unusual prescribing behavior in primary care data at multiple administrative levels in the NHS in England and to visualize these results using organization-specific interactive dashboards, thereby demonstrating proof of concept for prioritization approaches.

**Methods:**

Here we report a new data-driven approach to quantify how “unusual” the prescribing rates of a particular chemical within an organization are as compared to peer organizations, over a period of 6 months (June-December 2021). This is followed by a ranking to identify which chemicals are the most notable outliers in each organization. These outlying chemicals are calculated for all practices, primary care networks, clinical commissioning groups, and sustainability and transformation partnerships in England. Our results are presented via organization-specific interactive dashboards, the iterative development of which has been informed by user feedback.

**Results:**

We developed interactive dashboards for every practice (n=6476) in England, highlighting the unusual prescribing of 2369 chemicals (dashboards are also provided for 42 sustainability and transformation partnerships, 106 clinical commissioning groups, and 1257 primary care networks). User feedback and internal review of case studies demonstrate that our methodology identifies prescribing behavior that sometimes warrants further investigation or is a known issue.

**Conclusions:**

Data-driven approaches have the potential to overcome existing biases with regard to the planning and execution of audits, interventions, and policy making within NHS organizations, potentially revealing new targets for improved health care service delivery. We present our dashboards as a proof of concept for generating candidate lists to aid expert users in their interpretation of prescribing data and prioritize further investigations and qualitative research in terms of potential targets for improved performance.

## Introduction

There is recognition that evidence-based decision-making in the National Health Service (NHS) in England is critical to maintaining standards of care while reducing NHS spending [1] and the UK government has recently consulted on wide-ranging plans to “digitize, connect, and transform the health and care sector,” with a key priority being data-driven innovation. Flagship initiatives such as Getting It Right First Time [2] and RightCare [3] focus on identifying and addressing unwarranted variation in the NHS. Such initiatives can be limited in their scope in that the “data-driven” element of the work often focuses on assessing performance relative to recommendations that are defined prospectively rather than employing hypothesis-free data-driven methodologies to make objective assessments as to where opportunities for improvement might exist.

Monthly prescription data for every general practice in England has been made available to the public since 2010, via the NHS Business Services Authority [4]. This data set includes product and month of prescribing, the number of items prescribed and the total quantity, making it very amenable to detailed analysis for the purposes of original research [5-9] and systematic audits and reviews [10,11]. These data, made navigable via interactive dashboards [12,13], are commonly used by NHS staff—in particular, medicines optimization (MO) teams—to monitor performance on key prescribing indicators, compare performance to peer organizations, inform prioritization of work streams, estimate the impact and feasibility of interventions, or create customized outputs according to local priorities. Mining these data systematically for unusual prescribing behavior could help identify where service delivery improvements are possible in the absence of human bias or expectation. Such “unbiased” or “hypothesis-free” approaches might aid local decision makers when designing appropriate interventions and policies.

The value of deploying systematic analyses to large prescribing data sets has been demonstrated elsewhere. Using regional prescribing claims data from Germany, researchers were able to identify practices prescribing more “third-level” medications (ie, not first- or second-line treatments) than expected using funnel plots and mixed effects models [14]. Our own group has successfully deployed similar outlier detection methodology on a national scale to show that the prescribing of 2 antipsychotic drugs, in very limited use nationally, is concentrated in 2 small geographic regions of England [15]. More complex outlier analysis of wholesale codeine time series data has identified significant shifts in supply occurring around the time of regulatory changes (specifically, the up-scheduling of low-dose codeine products from over-the-counter to prescription-only) [16].

We run OpenPrescribing [13], a website that allows public interrogation and visualization of primary care prescription data at multiple administrative levels in the NHS in England. We have previously deployed novel methodologies to identify changes over time in any one of the 80 measures implemented in OpenPrescribing, providing monthly alerts to notify practitioners when their prescribing rates deviate from the norm and may require clinician attention [17]. These measures have been selected on the basis of clear guidance being available from health authorities and are subject to initial and continuing review by clinicians, pharmacists, and epidemiologists. OpenPrescribing has 20,000 unique users every month and thousands of subscribers to our innovative organization email alerts service [17].

We set out to develop new hypothesis-blind data science techniques to identify unusual prescribing behavior, thereby providing proof of concept for such an analysis and illustrating potential opportunities for service improvement. Using this approach, we have no hypothesis with regard to where interesting patterns might be found (ie, which clinical area or which organization), we only have an expectation of what would constitute an interesting pattern in the data. We applied this methodology to 6 months of national prescribing data to identify outliers at multiple administrative levels of the NHS in England during that time period, presenting the most extreme outliers in each organization for the consideration of expert users to prioritize for further review, qualitative research, and interpretation within the local context.

## Methods

### Study Design

Prescribing practice was analyzed by conducting a retrospective cohort study using prescribing data from all English NHS general practices, primary care networks (PCNs), clinical commissioning groups (CCGs), and sustainability and transformation partnership (STPs; Textbox 1).

**National Health Service (NHS) England administrative organizations.**

- Primary care in England is delivered by individual general practices, with one or more general practitioners. Almost all (>99%) practices are grouped together with other local primary care provision to form primary care networks, typically representing 30,000-50,000 people [18]. In the study period, practices were also grouped together into Clinical Commissioning Groups (CCGs) which were clinically-led organizations responsible for the commission of primary (and secondary) care in a geographical region [19]. As of April 2021, there were 106 CCGs in England. In the study period, CCGs were clustered into Sustainability and Transformation Partnerships (STPs) [20]. As of May 2020, there were 42 STPs in England. In July 2022, CCGs and STPs were replaced with 42 integrated care boards (ICBs), though the data used in this study predates this change.
- Also important in this architecture are the medicines optimization teams—NHS staff who provide expert advice with regard to medicines commissioning, finance, and safety [21]. Medicines optimization teams have historically operated at the level of CCGs (or their sub-ICB replacements) but are increasingly operating at the broader level of ICBs.

### Data Source

Data for the period June 1, 2021, to December 1, 2021, were extracted from the OpenPrescribing database; this 6-month study period was used so as to smooth out short-term fluctuations (by aggregating multiple months of data) while keeping to a relatively recent time frame (so that the data remain relevant). OpenPrescribing imports openly accessible prescribing data from the large, monthly files published by the NHS Business Services Authority, which contain data on cost and items prescribed for each month for every typical general practice and CCG in England, dating back to mid-2010 [4,22]. These data are published only at the level of organization; patient-level data are not made available. Detailed methods for the creation of OpenPrescribing, including data management, aggregation, and cleaning, are available elsewhere [23]. The monthly prescribing data sets contain 1 row for each different medication and dose in each prescribing organization in NHS primary care in England, describing the number of items (ie, prescriptions issued) and the total cost. These data are sourced from community pharmacy claims data and, therefore, contain all items that were dispensed. All available prescribing data were extracted for institutions identified as “typical” general practices; all other organizations, such as prisons or specialist community clinics, were excluded using NHS Digital organization data [24]. We limited our analysis to the 2369 chemicals from chapters 1-15 of the British National Formulary (BNF) to exclude chapters not following a chemical and subparagraph structure, those which largely cover nonmedicinal products such as dressings (see Textbox 2 for further information regarding prescribing terminology).

**Prescribing terminology.**

- The public prescribing data made available by the National Health Service Business Services Authority uses a pseudo-British National Formulary (BNF) classification. The most granular level of data is at “presentation” level, which includes information on the prescription medicine, brand, strength, and formulation. This data can then be grouped using the pseudo-BNF hierarchy, using products, chemical substances, subparagraphs, paragraphs, sections, and chapters, with decreasing specificity. Chapters are defined according to body system, for example, gastrointestinal system, cardiovascular system, and respiratory system.
- “Chemical” in this context refers to the standard International Nonproprietary Name (INN) for the active constituent of the medicine and does not include any further specification by preparation, dose, or brand. BNF subparagraphs can be used to identify groups of chemicals belonging to the same class.
- The majority of chemicals have all available preparations included in a single chemical definition; for example, all atorvastatin preparations (including liquid and tablets) are included in 2.12: Cardiovascular system—lipid-regulating drugs (chemical code: 0212000B0).
- However, there are some instances where the same chemical is used in different body systems with system-specific presentations, and therefore the same INN will appear multiple times in a chapter that most reflects its use. For example, the INN dexamethasone appears in 3 separate chapters within the pseudo-BNF hierarchy and therefore will have separate chemical groupings:

*6.3: Endocrine system—corticosteroids (endocrine)*, which include oral and parenteral preparations (chemical code: 0603020G0)

*11.4: Eye—corticosteroids and other anti-inflammatory preparations*, which include ocular preparations (chemical code: 1104010I0)

*12.1 Ear, Nose, and Oropharynx—drugs acting on the ear*, which ear preparations (chemical code: 12101050)

### Outlier Detection

We were interested in detecting outliers with regard to *chemicals* (see Textbox 2 for further information). We first calculate a prescription rate for each chemical in each practice; specifically, we calculate the number of prescriptions containing our chemical of interest and divide this by the number of prescriptions containing chemicals of the same BNF subparagraph, for example, all statin prescriptions as a proportion of all lipid-regulating drugs. This captures the prescribing rate for the chemical of interest as compared to all drugs in the same class in a single practice. This ratio is calculated across all practices, and the mean and SD are calculated. The ratios in each practice are then reexpressed as *z* scores using this mean and SD. A *z* score is the number of SDs that a given data point is away from the mean. The *z* scores are used to rank all chemicals within a practice in terms of their outlier status (the most extreme outliers occupying the top and bottom of this ranked list).

This process is repeated at 3 higher administrative levels—STP, CCG, and PCN—to generate the equivalent ranked list of prescribing outliers for these larger organizations. Results for all 4 administrative levels are presented, as each organization retains some decision-making power with regard to prescribing. At the practice or PCN level this will be individual or group general practitioner decisions based on their practice population, but MO teams at the STP and CCG level will also monitor prescribing behavior to inform prescribing policy (and formulary) for these wider geographic regions.

### Visualization of Organization-Level Results

An interactive dashboard has been created at OpenPrescribing [25] for each organization, where data describing 20 of the most extreme outliers are summarized as follows: 10 where prescribing in the organization is *higher* than other peer organizations, and 10 where prescribing in the organization is *lower* than other peer organizations. Tables are provided for both sets, which summarize the following values for each chemical: *Chemical Items* and *Subparagraph Items* are the number of prescriptions for the chemical and BNF subparagraph, respectively; *Ratio* is the *Chemical Items* as a proportion of *Subparagraph Items* for the chemical in the organization of interest; *Mean* and *SD* summarize this ratio over all organizations; and *z score* is the *Ratio* reexpressed as a *z* score. This same information is described visually by a density plot (provided in the Multimedia Appendices), where the distribution of ratios across all organizations is captured by a blue line, with the ratio for the organization of interest indicated by a vertical red line. Densities are generated using the Seaborn kdeplot() function, setting the bandwidth for smoothing as suggested by Scott [26].

### User Feedback

Links to early prototypes were shared directly with a group of interested clinicians and pharmacists by email. Any feedback gained was used to inform the iterative development of the tool and proposed visualizations of the results. Further to this, the tool was shared more widely (via Twitter), and formal feedback was collected via a Google form (Textbox 3). Additional unstructured feedback was compiled from direct emails and mentions on social media.

**Outlier detection feedback form.**

**Respondent details:**

- Email. Free text
- Which organization’s report are you giving feedback on? Free text
- Please describe your relationship to the organization (eg, doctor, practice nurse, or commissioner). Free text

**Understandability:**

- Does this report make sense to you? Yes or No
- Any further comments on the understandability of the report(s). Free text

**Interest:**

- Is it interesting? Yes or No
- Any further comments on the interestingness of the report(s). Free text

**Utility:**

- Is it useful? Yes or No
- Any further comments on the usefulness of the report(s). Free text

**Individual items:**

- Thinking about where your prescribing is higher than most, please describe any observations you have on any individual items. Free text
- Thinking about where your prescribing is lower than most, please describe any observations you have on any individual items. Free text

**Improvements:**

- What, if anything, would you change about the report(s)? Free text

### NHS Devon CCG Case Study Details

RC (who, in addition to his role at the Bennett Institute, is also Deputy Director for MO at NHS Devon) emailed a link to the dashboard containing sparkline graphs for NHS Devon to pharmacist colleagues in his MO team. These graphs provided new insights to the team, which would have been impractical to achieve using existing data analysis workflows (eg, custom queries in OpenPrescribing or ePACT2). The MO team met to discuss what the causes behind the deviation might be in each case. Where it could not be determined that there was a clinically justifiable reason for being an outlier, the MO team gathered further relevant prescribing data from routine sources such as OpenPrescribing, ePACT2, and PrescQIPP. This allowed deeper exploration of prescribing patterns related to the outlier chemical (eg, trends over time and the rate at which alternative medications were prescribed). The MO team continues to investigate these data to decide whether an intervention is appropriate.

### Software and Reproducibility

Data management was performed using Python 3.8.1 and Google BigQuery, with analysis carried out using Python. Code for data management and analysis is archived on the internet [27] and dashboards are available on the OpenPrescribing website [25].

### Patient and Public Involvement

We publicized this tool via social media and actively sought feedback from interested health care professionals and members of the public to inform its iterative development via a survey (see User Feedback section above). We will continue to seek and consider feedback via these same channels as the tool is developed. We have developed a publicly available website [13] through which we invite any patient or member of the public to contact us regarding this study or the broader OpenPrescribing project.

## Results

### Outlier Detection

We developed interactive dashboards for every practice in England to highlight unusual prescribing. The outlying chemicals (ie, the 10 chemicals ranked highest and 10 chemicals ranked lowest by *z* score) identified using our methodology are described in Table 1. Both counts of unique chemicals and summary statistics of *z* scores are provided at each of the 4 administrative levels. Those outlying chemicals that are “higher than most” will all have positive *z* and as such are summarized using the maximum, median, Q1 and Q3; similarly, outlying chemicals that are “lower than most” will have negative *z* scores, and are summarized using the minimum, median, Q1 and Q3. A measure of the variation in the *z* score amongst all organizations at the same administrative level can be obtained by calculating the Inter Quartile Range (IQR), defined as Q3-Q1.

**Table 1:** Summary statistics for z scores calculated for outlying chemicals across the 4 administrative levels. Outlying chemicals are those occurring in the top 10 (ie, “Higher than most”) or bottom 10 (ie, “Lower than most”) by z score in at least one organization at the corresponding administrative level.

Organization type	Unique chemicals, n	Higher than most				Lower than most		
		Maximum	Median	Q1-Q3	Minimum		Median	Q1-Q3
STP^a^ (n=42)	680	6.33	5.42	4.6-6.24	−6.33		−2.35	−2.87-−2.08
CCG^b^ (n=106)	1138	10.20	5.79	4.59-7.77	−10.20		−2.30	−2.81-−1.99
PCN^c^ (n=1257)	1416	2528.09	5.28	4.17-7.56	−159.77		−2.18	−2.67-−1.9
Practice (n=6476)	1346	6825.50	5.23	3.93-7.77	−307.23		−2.08	−2.57-−1.76

^a^STP: sustainability and transformation partnership.

^b^CCG: clinical commissioning group.

^c^PCN: primary care network.

While the median values for the “higher than most” outlying chemicals are similar, the IQR (Q3-Q1) values demonstrate that variation between peer organizations decreases with the size of the organization; the least amount of variation is observed between STPs, and the most amount of variation is observed between practices. More outlying chemicals are identified in smaller organizations (PCNs and practices). With regard to outlying chemicals identified as being prescribed at lower rates compared to peer organizations, both the median and IQR of the *z* scores are very similar across all organization types. For both sets of outlying chemicals, the most extreme outliers occur further away from the mean as the organization size decreases; the maximum value for the “higher than most” outlying chemicals *increases* with the size of the organization and the minimum value for the “lower than most” outlying chemicals *decreases* with the size of the organization. The *z* scores for “higher than most” outlying chemicals are more extreme than the “lower than most” outlying chemicals in all organization types.

### Organization-Level Results Visualization: Case Study of NHS Devon CCG

NHS Devon CCG is the fifth largest CCG in England, commissioning health care for 1.2 million people in the southwest of England. The top 10 chemicals that are prescribed at *higher* rates here compared to other CCGs are shown in the top portion of Table 2, while the top 10 chemicals that are prescribed at *lower* rates are shown in the bottom portion of Table 2 (a listing of the specific products and a sparkline plot, showing graphically where the ratio value for this CCG occurs in the context of the same ratio in all CCGs, are provided in Multimedia Appendix 1). These prescribing outliers for this CCG have been reviewed by the local MO team, to provide likely explanations for the outlier prescribing.

**Table 2:** The outlier detection dashboard for NHS^a^ Devon clinical commissioning group (CCG)^b^.

BNF^c^ chemical (number of products)		Chemical items, n	BNF subparagraph	Subparagraph items, n	Ratio	Mean	SD	*z* score
**Prescribing where NHS** **Devon CCG is *higher* than most**								
	Levobupivacaine hydrochloride (1)	130	Local anesthetics	20,482	0.01	0	0	10.2
	Gripe mixtures (1)	1	Sodium bicarbonate	56	0.02	0	0	8.34
	Gluten free pastas (3)	4	Foods for special diets	9199	0	0	0	7.97
	Epoetin zeta (1)	2	Hypoplastic, hemolytic, and renal anemias	18	0.11	0	0.01	7.52
	Flumetasone pivalate (1)	333	Otitis externa	19,724	0.02	0	0	6.98
	Gluten free or wheat free cereals (1)	2	Foods for special diets	9199	0	0	0	6.81
	Levofloxacin (2)	1372	Quinolones	4754	0.29	0.05	0.04	6.61
	Liquefied phenol (1)	1	Phenolics	3	0.33	0.01	0.06	5.83
	Ruxolitinib (1)	2	Other antineoplastic drugs	1494	0	0	0	5.10
	Ferrous gluconate (1)	10,437	Oral iron	90,095	0.12	0.03	0.02	3.66
**Prescribing where NHS Devon CCG is *lower* than most**								
	Sodium bicarbonate (3)	55	Sodium bicarbonate	56	0.98	1	0	–8.34
	Ciprofloxacin (6)	2989	Quinolones	4754	0.63	0.86	0.05	–4.22
	Dexamethasone (2)	13,061	Otitis externa	19,724	0.66	0.79	0.05	–2.71
	Fexofenadine hydrochloride (6)	33,711	Antihistamines	169,747	0.20	0.36	0.07	–2.33
	Oral rehydration salts (8)	1942	Oral sodium and water	5010	0.39	0.65	0.11	–2.27
	Betamethasone esters (12)	1672	Topical corticosteroids	141,063	0.01	0.03	0.01	–2.20
	Fusidic acid (1)	359	Antibacterials	13,283	0.03	0.08	0.03	–2.12
	Senna (9)	39,769	Stimulant laxatives	110,838	0.36	0.55	0.1	–2.03
	Ticagrelor (3)	2285	Antiplatelet drugs	467,104	0	0.02	0.01	–2.02
	Lactulose (2)	19,621	Osmotic laxatives	127,773	0.15	0.28	0.06	–1.89

^a^NHS: National Health Service.

^b^"The results of our outlier detection methodology are provided as interactive dashboards; here, the 10 chemicals where prescribing in National Health Service (NHS) Devon CCG is higher than most and the 10 chemicals where prescribing in NHS Devon CCG is lower than most, are presented. British National Formulary (BNF) chemical is the chemical of interest (number of products indicates how many products are represented by the BNF chemical). Chemical items provide the number of prescribing items containing this chemical. BNF subparagraph is the BNF subparagraph to which the chemical belongs, and subparagraph items is the number of prescribing items containing an item belonging to this BNF subparagraph. Ratio, Mean, SD, and *z* score place the chemical items count in the context of the subparagraph items count as described in the Methods section.

^c^BNF: British National Formulary.

Focusing on the results for flumetasone pivalate, we can see that 1.7% (n=333) of the 19,724 “Otitis externa” items contain flumetasone pivalate and that this is 6.98 SDs above the mean for all CCGs (the sparkline plot provided in Multimedia Appendix 1 demonstrates visually where this 1.7% falls [red line] in the distribution across all CCGs [blue line]).

Several of the chemicals prescribed more often in NHS Devon CCGs than other CCGs are defined as first-line treatments in local formularies, for example, flumetasone pivalate [28] and levofloxacin [29]. Corresponding patterns of underprescribing can be seen in the “lower than most” results table for similar chemicals, specifically, ciprofloxacin (an alternative to levofloxacin) and dexamethasone (an alternative to flumetasone pivalate).

The lower prescribing rates for fusidic acid reflect a change in this CCG to prescribe this chemical by specialist recommendation only [30], due to rising costs [31] and a narrow spectrum of action. The lower rates of prescribing for senna and lactulose are also likely due to a formulary shift in this CCG toward macrogols [32]. Finally, the low prescribing rate of betamethasone esters is also expected as these chemicals are nonformulary in this CCG [33].

This dashboard also demonstrates a valid use for low-number results. Gluten-free pastas and cereals—something that we have previously identified as having high variability in prescribing rates [34]—were not recommended to be prescribed by the NHS in the study period (NHS England issued advice to CCGs in November 2018 with the recommendation to restrict gluten free prescribing to bread and flour mixes [35]), so should not appear at all. The identification of this low-number outlier via our methodology has prompted further work within NHS Devon CCG to clarify how this prescription was generated and processed.

### User Feedback

Through the formal Google form and direct correspondence with interested parties, we received feedback for a prototype version of the dashboard from 6 individuals. An example of this prototype is shown in Multimedia Appendix 2, showing 5 top and bottom outlying chemicals. Several respondents indicated that the results were expected (ie, results echoed internal reporting or were aligned with local prescribing policies); while this indicates that our tool is working, 1 user did question what the added value was above existing reporting. Other users stated that the tool had revealed unexpected results worthy of follow-up.

There were multiple requests to present more than the top and bottom 5 results (eg, the top and bottom 10 or 20 results) to explore the data in more detail. Users recognized that extreme outliers could be derived from very small numbers of patients or items; some requested that results with small counts be removed, though others recognized that these may be important, particularly in practices or PCNs. There was a suggestion that users could choose to have low numbers suppressed or displayed, depending on whether their focus was systemic anomalies or rogue prescriptions. There were also requests to include other data in the results, including cost and highlighting drugs on the “*Not Suitable to Prescribe*” list.

There were other requests that were more relevant to the design of the tool than the analysis itself. The feedback demonstrated that users required more information to interpret and understand the data (ie, *z* scores, ratios, means, and SDs) and that with this additional explanation, more could be made of the graphical summary. There was also a request for an improved user experience regarding navigating to practices via the drop-down sections (which could be implemented as an organizational search).

We used the most common feedback to inform further development, and the released version of the dashboards now includes the top and bottom 10 outlying chemicals and optional filtering of low numbers. To provide a clear illustration of how the dashboards changed in response to user feedback, the corresponding update for Multimedia Appendix 2 is shown in Figures S1 and S2 in Multimedia Appendix 3.

## Discussion

### Summary

We have developed and implemented a new hypothesis-free methodology to detect unusual or “outlier” prescribing rates of chemicals in a single organization in relation to all “peer” organizations. We have applied this methodology to 6 months of national prescribing data to quantify how typical the prescribing is for individual chemicals at multiple administrative levels (practice, PCN, CCG, and STP) over the time period. We have displayed these results via interactive dashboards. We have sought and will continue to seek user feedback to inform development and incrementally improve usability and functionality.

Summary statistics demonstrate that the number of outlying chemicals increases as the size of the organization decreases and that more extreme outliers are identified among smaller organizations, demonstrating that there is more variability in prescribing behavior among practices than there is among larger administrative organizations. The data also demonstrate, however, that outliers *do* occur when comparing larger organizations to each other. While there is less variation between STPs, the median *z* score for “higher than most” and “lower than most” outliers among STPs is 5.42 and 2.35, respectively; these *z* scores are both more than 2 SDs from the mean. The ranking of these quantifications allows us to identify the most extreme outliers in terms of prescribing behavior at each organizational level. A case study of an individual CCG (NHS Devon) demonstrated that our methodology identified prescribing patterns that aligned with local prescribing guidance, but also detected patterns that warranted further investigation. It is not appropriate to formally assess the utility of our methodology as there are many legitimate reasons that a chemical may be an outlier in a particular organization. Some of the reasons are as follows: prescribing guidance as defined by local formulary may differ from elsewhere; local prescribing policy may place responsibility for prescribing particular drugs in secondary care rather than primary care; clinicians may be reluctant to change medication for patients who are stable on a long-established medication regime (in particular the elderly or vulnerable); or there is a justified preference for other drugs in the same class. Given the complexities of interpreting these data, we present this tool as a proof of concept and starting point for NHS organizations to perform and plan internal audits rather than a definitive reporting tool.

### Strengths and Weaknesses

Our approach combines a comprehensive national prescribing data set with a well-understood system for drug classification, thereby capturing the national context at high resolution and allowing the interpretation of prescribing behavior for *all* chemicals at multiple administrative levels of the NHS in England, all of which retain some decision-making power with regard to prescribing. The methods used are well established and easy to understand, readily amenable to visual presentation as graphs, and allow prioritization of results by ranking. Our approach has utility in other contexts, and repurposing it to gain a greater understanding of other NHS data (eg, hospital prescriptions) would be straightforward.

We also note some limitations. First, the calculation of *z* scores using mean and SD assumes a normal distribution. This is more likely to be the case where numbers of items prescribed are high (aggregated to STP or CCG), but may not be the case where number of items are low (aggregated to PCN or practices, or where the items are more rarely prescribed). Second, this approach can generate very large *z* scores where SDs are tight or item numbers generally are very low. An example of this can be seen in Figure S1 in Multimedia Appendix 3; while the ratio generated by the number of prescribed items containing Sodium aurothiomalate is very low (1/114,367=8.74×10^6^), the tight SDs observed across the whole population of STPs translate this small value into a large *z* score. Expert users may be seeking out such results to identify very rare prescribing items (low number results did prove important in the NHS Devon case study), but they may also wish to suppress such results to focus on more commonly prescribed chemicals. To accommodate this and in line with our user feedback, we have implemented the option to show or hide counts of 5 or less. We also recognize that the process by which we have sought user feedback thus far could be prone to bias, in that specific users were targeted due to their expertise and familiarity with such tools so as to enable rapid development.

### Findings in Context

This is one of a suite of tools that we are seeking to develop at OpenPrescribing, each of which captures variability with a view to leveraging further insight from the data sets to which we have access. We make extensive use of decile plots to place individual organizations into a wider context [5,6,36] and have applied algorithms to identify when those individual organizations start to deviate from the rest of their peers [17]. We have also used deciles to summarize financial data and estimate potential savings if “price-per-unit” costs were aligned with the lowest decile [7]. These methodologies all have the potential to support NHS organizations in England to guide audits, prioritize and shape new policies, and crucially assess the impact of those interventions with regard to patient care and cost savings.

### Policy Implications and Interpretation

The Department of Health and Social Care consultation explicitly recognizes the value of near real-time data release and the potential of data-driven insights to guide targeted policy making [37]. The methodology described here contributes toward that key priority by exposing specific patterns in data that warrant attention that may have otherwise been obscured. We do not advocate that our approach be used in isolation, but rather as a starting point for expert users to interpret within the local context and make evidence-based decisions about priorities and planning. By updating these dashboards on a regular basis, we hope to provide decision makers with near real-time feedback so as to monitor performance and respond quickly when necessary. Comprehensive coverage of the opportunities and challenges that exist in encouraging widespread adoption of these approaches across the NHS in England is provided in the Goldacre Review [38].

### Future Research

Areas for further research include the implementation of a systematic and unbiased approach to collecting and inviting user feedback, enhancing results output as determined by ongoing user feedback (eg, new functionality, information, or visualizations), updating the dashboards in line with recent structural changes to the NHS in England (specifically, Integrated Care Boards replacing STPs), and consulting with patient and public involvement and engagement groups to maximize value for the patient community. The long-term aim is to incorporate regular updates as part of an organization’s page on the OpenPrescribing website; the frequency of these updates (annual vs monthly) and the extent to which historical dashboards would be available for each organization have yet to be determined but would be a focus of the enhanced user consultation described above. Ultimately, our aim would be to provide organization specific alerts to notify staff where prescribing behavior appears to be different to their peers.

### Conclusions

Capturing the variability in prescribing rates among peer organizations permits the hypothesis-free identification of prescribing outliers. We have applied such an analysis to 6 months of national prescribing data and made the most extreme prescribing outliers in each organization publicly available as interactive dashboards. We intend that these dashboards prompt further qualitative analysis within the individual organizations to identify where service delivery improvements could be made.

## Appendix

Multimedia Appendix 1 The outlier detection dashboard for Devon CCG (including product listings and sparkline plots). The results of our outlier detection methodology are provided as interactive dashboards; here the ten chemicals where prescribing in NHS Devon CCG is higher than most and the ten chemicals where prescribing in NHS Devon CCG is lower than most are presented. Data for each result is highlighted in grey with additional information provided below with no highlighting. BNF Chemical is the chemical of interest (all products represented by this BNF chemical are provided as additional information). Chemical Items provides the number of prescribing items containing this chemical. BNF Subparagraph is the BNF Subparagraph to which the Chemical belongs and Subparagraph Items is the number of prescribing items containing an item belonging to this BNF Subparagraph. Ratio, Mean, std and Z-score place the chemical items count in the context of the subparagraph items count as described in the methods. The sparkline plot provided as additional information for each result shows where the Ratio value for this CCG occurs (vertical red line) in the context of the same Ratio in all CCGs (summarised by the blue line). The y axis is density (see Methods).

Multimedia Appendix 2 Prototype dashboard showing the top and bottom five outlying chemicals for Cumbria and northeast STP. BNF Chemical is the chemical of interest, Chemical Items provides the number of prescribing items containing this chemical. BNF Subparagraph is the BNF Subparagraph to which the Chemical belongs and Subparagraph Items is the number of prescribing items containing an item belonging to this BNF Subparagraph. Ratio, Mean, std, and Z_Score place the chemical items count in the context of the subparagraph items count as described in the methods. The sparkline plot shows where the ratio value for this STP occurs (vertical red line) in the context of the same ratio in all STPs (summarised by the blue line).

Multimedia Appendix 3 Example dashboard showing the top ten outlying chemicals for Cumbria and northeast STP. BNF Chemical is the chemical of interest, Chemical Items provides the number of prescribing items containing this chemical. BNF Subparagraph is the BNF subparagraph to which the chemical belongs and Subparagraph Items is the number of prescribing items containing an item belonging to this BNF Subparagraph. Ratio, Mean, std, and Z score place the chemical items count in the context of the subparagraph items count as described in the methods. The sparkline plot shows where the ratio value for this STP occurs (vertical red line) in the context of the same Ratio in all STPs (summarised by the blue line). Example dashboard showing the bottom ten outlying chemicals for Cumbria and northeast STP. See Figure S1 for definitions of each column.

## Abbreviations

BNF: British National Formulary
CCG: clinical commissioning group
MO: medicines optimization
NHS: National Health Service
PCN: primary care network
STP: sustainability and transformation partnership
