# Supplementary material for: Data-Driven Identification of Unusual Prescribing Behavior: Analysis and Use of an Interactive Data Tool Using 6 Months of Primary Care Data From 6500 Practices in England
Source: JMIR Med Inform. 2023 Apr 19;11:e44237. doi: 10.2196/44237 (PMC10162592; doi:10.2196/44237)
Supplement: Multimedia Appendix 1 [file medinform_v11i1e44237_app1.docx]

**Multimedia Appendix 1**: The outlier detection dashboard for Devon CCG (including product listings and sparkline plots). The results of our outlier detection methodology are provided as interactive dashboards; here, the ten chemicals where prescribing in NHS Devon CCG is *higher* than most and the ten chemicals where prescribing in NHS Devon CCG is *lower* than most, are presented. Data for each result is highlighted in grey with additional information provided below with no highlighting. BNF Chemical is the chemical of interest (all products represented by this BNF chemical are provided as additional information). Chemical Items provides the number of prescribing items containing this chemical. BNF Subparagraph is the BNF Subparagraph to which the Chemical belongs and Subparagraph Items is the number of prescribing items containing an item belonging to this BNF Subparagraph. Ratio, Mean, std and Z-score place the chemical items count in the context of the subparagraph items count as described in the methods. The sparkline plot provided as additional information for each result shows where the Ratio value for this CCG occurs (vertical red line) in the context of the same Ratio in all CCGs (summarised by the blue line). The y axis is density (see Methods).

| **BNF Chemical** | **Chemical Items** | **BNF Subparagraph** | **Subparagraph Items** | **Ratio** | **Mean** | **std** | **Z-Score** |
| --- | --- | --- | --- | --- | --- | --- | --- |
| Prescribing where NHS Devon CCG is *higher* than most | | | | | | | |
| [Levobupivacaine hydrochloride](https://openprescribing.net/analyse/#org=practice&orgIds=15N&numIds=1502010V0&denomIds=15.2.1&selectedTab=summary) | 130 | Local anaesthetics | 20,482 | 0.01 | 0.00 | 0.00 | 10.20 |
| [Chirocaine 25mg/10ml solution for injection ampoules : 130.000000000](https://openprescribing.net/analyse/#org=practice&orgIds=15N&numIds=1502010V0BBAAAA&denomIds=15.2.1&selectedTab=summary) | | | | 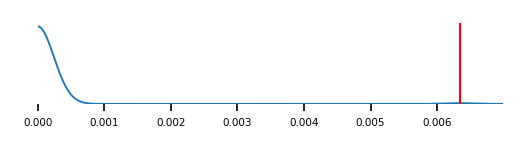 | | | |
| [Gripe mixtures](https://openprescribing.net/analyse/#org=practice&orgIds=15N&numIds=0101012A0&denomIds=1.1.1&selectedTab=summary) | 1 | Sodium bicarbonate | 56 | 0.02 | 0.00 | 0.00 | 8.34 |
| [Woodward's gripe water : 1.000000000](https://openprescribing.net/analyse/#org=practice&orgIds=15N&numIds=0101012A0BBAAAA&denomIds=1.1.1&selectedTab=summary) | | | | 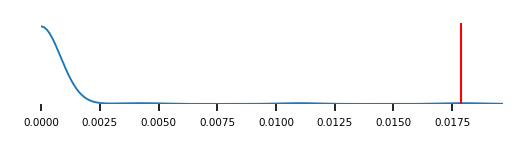 | | | |
| [Gluten free pastas](https://openprescribing.net/analyse/#org=practice&orgIds=15N&numIds=0904010Q0&denomIds=9.4.1&selectedTab=summary) | 4 | Foods for special diets | 9,199 | 0.00 | 0.00 | 0.00 | 7.97 |
| [Glutafin gluten free pasta fibre fusilli : 2.000000000](https://openprescribing.net/analyse/#org=practice&orgIds=15N&numIds=0904010Q0BLABAQ&denomIds=9.4.1&selectedTab=summary)  [Juvela gluten free pasta fusilli : 1.000000000](https://openprescribing.net/analyse/#org=practice&orgIds=15N&numIds=0904010Q0BRACAQ&denomIds=9.4.1&selectedTab=summary)  [Juvela gluten free pasta spaghetti : 1.000000000](https://openprescribing.net/analyse/#org=practice&orgIds=15N&numIds=0904010Q0BRAFAQ&denomIds=9.4.1&selectedTab=summary) | | | | 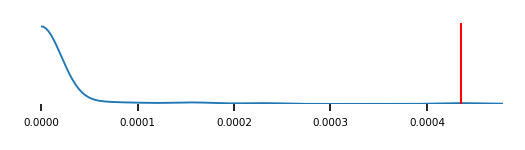 | | | |
| [Epoetin zeta](https://openprescribing.net/analyse/#org=practice&orgIds=15N&numIds=0901030Q0&denomIds=9.1.3&selectedTab=summary) | 2 | Hypoplastic/haemolytic and renal anaemias | 18 | 0.11 | 0.00 | 0.01 | 7.52 |
| [Epoetin zeta 4,000units/0.4ml inj pre-filled syringes : 2.000000000](https://openprescribing.net/analyse/#org=practice&orgIds=15N&numIds=0901030Q0AAADAD&denomIds=9.1.3&selectedTab=summary) | | | | 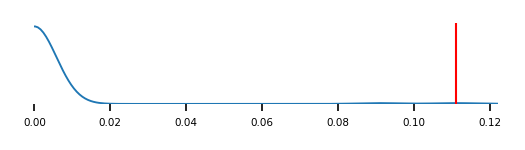 | | | |
| [Flumetasone pivalate](https://openprescribing.net/analyse/#org=practice&orgIds=15N&numIds=1201010F0&denomIds=12.1.1&selectedTab=summary) | 333 | Otitis externa | 19,724 | 0.02 | 0.00 | 0.00 | 6.98 |
| [Flumetasone 0.02% / Clioquinol 1% ear drops : 333.000000000](https://openprescribing.net/analyse/#org=practice&orgIds=15N&numIds=1201010F0AAAAAA&denomIds=12.1.1&selectedTab=summary) | | | | 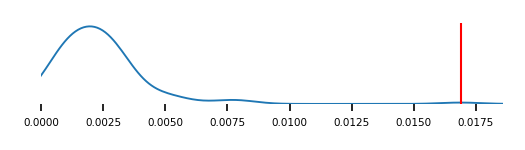 | | | |
| [Gluten free/wheat free cereals](https://openprescribing.net/analyse/#org=practice&orgIds=15N&numIds=0904010AC&denomIds=9.4.1&selectedTab=summary) | 2 | Foods for special diets | 9,199 | 0.00 | 0.00 | 0.00 | 6.81 |
| [Juvela gluten free flakes : 2.000000000](https://openprescribing.net/analyse/#org=practice&orgIds=15N&numIds=0904010ACBIAAAC&denomIds=9.4.1&selectedTab=summary) | | | | 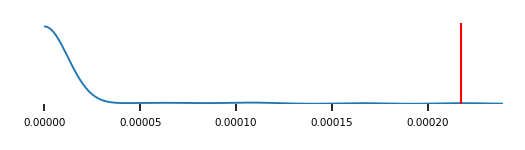 | | | |
| [Levofloxacin](https://openprescribing.net/analyse/#org=practice&orgIds=15N&numIds=0501120X0&denomIds=5.1.12&selectedTab=summary) | 1,372 | Quinolones | 4,754 | 0.29 | 0.05 | 0.04 | 6.61 |
| [Levofloxacin 500mg tablets : 1207.000000000](https://openprescribing.net/analyse/#org=practice&orgIds=15N&numIds=0501120X0AAAAAA&denomIds=5.1.12&selectedTab=summary)  [Levofloxacin 250mg tablets : 165.000000000](https://openprescribing.net/analyse/#org=practice&orgIds=15N&numIds=0501120X0AAABAB&denomIds=5.1.12&selectedTab=summary) | | | | 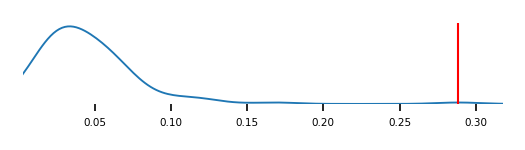 | | | |
| [Liquefied phenol](https://openprescribing.net/analyse/#org=practice&orgIds=15N&numIds=1311050P0&denomIds=13.11.5&selectedTab=summary) | 1 | Phenolics | 3 | 0.33 | 0.01 | 0.06 | 5.83 |
| [Phenol liquefied : 1.000000000](https://openprescribing.net/analyse/#org=practice&orgIds=15N&numIds=1311050P0AAABAB&denomIds=13.11.5&selectedTab=summary) | | | | 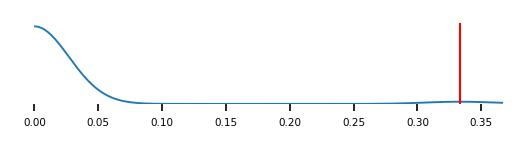 | | | |
| [Ruxolitinib](https://openprescribing.net/analyse/#org=practice&orgIds=15N&numIds=0801050BI&denomIds=8.1.5&selectedTab=summary) | 2 | Other antineoplastic drugs | 1,494 | 0.00 | 0.00 | 0.00 | 5.10 |
| [Ruxolitinib 10mg tablets : 2.000000000](https://openprescribing.net/analyse/#org=practice&orgIds=15N&numIds=0801050BIAAADAD&denomIds=8.1.5&selectedTab=summary) | | | | 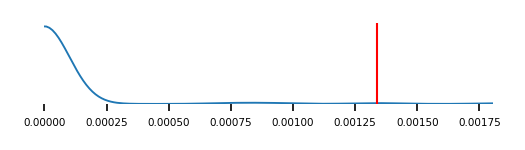 | | | |
| [Ferrous gluconate](https://openprescribing.net/analyse/#org=practice&orgIds=15N&numIds=0901011H0&denomIds=9.1.1&selectedTab=summary) | 10,437 | Oral iron | 90,095 | 0.12 | 0.03 | 0.02 | 3.66 |
| [Ferrous gluconate 300mg tablets : 10437.000000000](https://openprescribing.net/analyse/#org=practice&orgIds=15N&numIds=0901011H0AAAAAA&denomIds=9.1.1&selectedTab=summary) | | | | 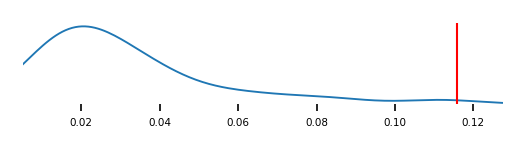 | | | |
| Prescribing where NHS Devon CCG is *lower*  than most | | | | | | | |
| [Sodium bicarbonate](https://openprescribing.net/analyse/#org=practice&orgIds=15N&numIds=0101012B0&denomIds=1.1.1&selectedTab=summary) | 55 | Sodium bicarbonate | 56 | 0.98 | 1.00 | 0.00 | -8.34 |
| [Sodium bicarbonate 420mg/5ml (1mol/ml) soln sugar free : 32.000000000](https://openprescribing.net/analyse/#org=practice&orgIds=15N&numIds=0101012B0AAABAB&denomIds=1.1.1&selectedTab=summary)  [Sodium bicarbonate powder : 15.000000000](https://openprescribing.net/analyse/#org=practice&orgIds=15N&numIds=0101012B0AAAPAP&denomIds=1.1.1&selectedTab=summary)  [Bidex 84mg/ml oral solution : 8.000000000](https://openprescribing.net/analyse/#org=practice&orgIds=15N&numIds=0101012B0BLAAAB&denomIds=1.1.1&selectedTab=summary) | | | | 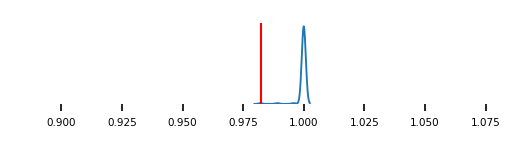 | | | |
| [Ciprofloxacin](https://openprescribing.net/analyse/#org=practice&orgIds=15N&numIds=0501120L0&denomIds=5.1.12&selectedTab=summary) | 2,989 | Quinolones | 4,754 | 0.63 | 0.86 | 0.05 | -4.22 |
| [Ciprofloxacin 250mg tablets : 671.000000000](https://openprescribing.net/analyse/#org=practice&orgIds=15N&numIds=0501120L0AAAAAA&denomIds=5.1.12&selectedTab=summary)  [Ciprofloxacin 500mg tablets : 2156.000000000](https://openprescribing.net/analyse/#org=practice&orgIds=15N&numIds=0501120L0AAAFAF&denomIds=5.1.12&selectedTab=summary)  [Ciprofloxacin 100mg tablets : 1.000000000](https://openprescribing.net/analyse/#org=practice&orgIds=15N&numIds=0501120L0AAAGAG&denomIds=5.1.12&selectedTab=summary)  [Ciprofloxacin 750mg tablets : 90.000000000](https://openprescribing.net/analyse/#org=practice&orgIds=15N&numIds=0501120L0AAAJAJ&denomIds=5.1.12&selectedTab=summary)  [Ciprofloxacin 250mg/5ml oral suspension : 70.000000000](https://openprescribing.net/analyse/#org=practice&orgIds=15N&numIds=0501120L0AABGBG&denomIds=5.1.12&selectedTab=summary)  [Ciproxin 500mg tablets : 1.000000000](https://openprescribing.net/analyse/#org=practice&orgIds=15N&numIds=0501120L0BBABAF&denomIds=5.1.12&selectedTab=summary) | | | | 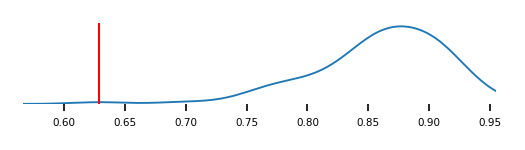 | | | |
| [Dexamethasone](https://openprescribing.net/analyse/#org=practice&orgIds=15N&numIds=120101050&denomIds=12.1.1&selectedTab=summary) | 13,061 | Otitis externa | 19,724 | 0.66 | 0.79 | 0.05 | -2.71 |
| [Generic Otomize ear spray : 2.000000000](https://openprescribing.net/analyse/#org=practice&orgIds=15N&numIds=120101050AAABAB&denomIds=12.1.1&selectedTab=summary)  [Otomize ear spray : 13059.000000000](https://openprescribing.net/analyse/#org=practice&orgIds=15N&numIds=120101050BCAAAB&denomIds=12.1.1&selectedTab=summary) | | | | 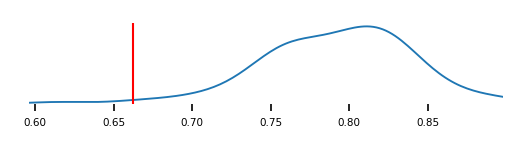 | | | |
| [Fexofenadine hydrochloride](https://openprescribing.net/analyse/#org=practice&orgIds=15N&numIds=0304010E0&denomIds=3.4.1&selectedTab=summary) | 33,711 | Antihistamines | 169,747 | 0.20 | 0.36 | 0.07 | -2.33 |
| [Fexofenadine 120mg tablets : 12548.000000000](https://openprescribing.net/analyse/#org=practice&orgIds=15N&numIds=0304010E0AAAAAA&denomIds=3.4.1&selectedTab=summary)  [Fexofenadine 180mg tablets : 20539.000000000](https://openprescribing.net/analyse/#org=practice&orgIds=15N&numIds=0304010E0AAABAB&denomIds=3.4.1&selectedTab=summary)  [Fexofenadine 30mg tablets : 445.000000000](https://openprescribing.net/analyse/#org=practice&orgIds=15N&numIds=0304010E0AAACAC&denomIds=3.4.1&selectedTab=summary)  [Fexofenadine 180mg/5ml oral liquid : 4.000000000](https://openprescribing.net/analyse/#org=practice&orgIds=15N&numIds=0304010E0AAAEAE&denomIds=3.4.1&selectedTab=summary)  [Telfast 120mg tablets : 59.000000000](https://openprescribing.net/analyse/#org=practice&orgIds=15N&numIds=0304010E0BBAAAA&denomIds=3.4.1&selectedTab=summary)  [Telfast 180mg tablets : 116.000000000](https://openprescribing.net/analyse/#org=practice&orgIds=15N&numIds=0304010E0BBABAB&denomIds=3.4.1&selectedTab=summary) | | | | 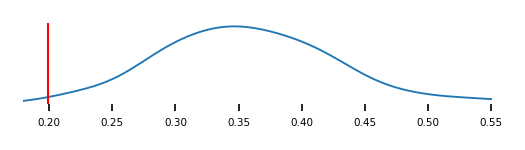 | | | |
| [Oral rehydration salts](https://openprescribing.net/analyse/#org=practice&orgIds=15N&numIds=0902012H0&denomIds=9.2.1&selectedTab=summary) | 1,942 | Oral sodium and water | 5,010 | 0.39 | 0.65 | 0.11 | -2.27 |
| [Generic Dioralyte oral powder sachets : 3.000000000](https://openprescribing.net/analyse/#org=practice&orgIds=15N&numIds=0902012H0AAAEAE&denomIds=9.2.1&selectedTab=summary)  [Glucose monohydrate 20g/Sod chlor 3.5g/Sod bicarb 2.5g pdr : 3.000000000](https://openprescribing.net/analyse/#org=practice&orgIds=15N&numIds=0902012H0AAAKAK&denomIds=9.2.1&selectedTab=summary)  [Dioralyte oral powder sachets plain : 629.000000000](https://openprescribing.net/analyse/#org=practice&orgIds=15N&numIds=0902012H0BBAEAE&denomIds=9.2.1&selectedTab=summary)  [Dioralyte oral powder sachets citrus : 189.000000000](https://openprescribing.net/analyse/#org=practice&orgIds=15N&numIds=0902012H0BBAFAE&denomIds=9.2.1&selectedTab=summary)  [Dioralyte oral powder sachets blackcurrant : 1084.000000000](https://openprescribing.net/analyse/#org=practice&orgIds=15N&numIds=0902012H0BBAGAE&denomIds=9.2.1&selectedTab=summary)  [Dioralyte Relief oral powder sachets raspberry : 6.000000000](https://openprescribing.net/analyse/#org=practice&orgIds=15N&numIds=0902012H0BBAKAJ&denomIds=9.2.1&selectedTab=summary)  [Dioralyte Relief oral powder sachets blackcurrant : 23.000000000](https://openprescribing.net/analyse/#org=practice&orgIds=15N&numIds=0902012H0BBALAJ&denomIds=9.2.1&selectedTab=summary)  [O.R.S Oral Rehydration Salts soluble tablets blackcurrant : 5.000000000](https://openprescribing.net/analyse/#org=practice&orgIds=15N&numIds=0902012H0BWABAN&denomIds=9.2.1&selectedTab=summary) | | | | 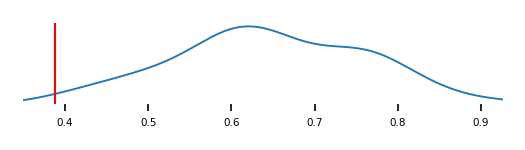 | | | |
| [Betamethasone esters](https://openprescribing.net/analyse/#org=practice&orgIds=15N&numIds=1304000D0&denomIds=13.4&selectedTab=summary) | 1,672 | Topical corticosteroids | 141,063 | 0.01 | 0.03 | 0.01 | -2.20 |
| [Betamethasone dipropionate 0.05% cream : 66.000000000](https://openprescribing.net/analyse/#org=practice&orgIds=15N&numIds=1304000D0AAAAAA&denomIds=13.4&selectedTab=summary)  [Betamethasone dipropionate 0.05% ointment : 36.000000000](https://openprescribing.net/analyse/#org=practice&orgIds=15N&numIds=1304000D0AABABA&denomIds=13.4&selectedTab=summary)  [Betamethasone dipropionate 0.05% / Salicylic acid 3% oint : 98.000000000](https://openprescribing.net/analyse/#org=practice&orgIds=15N&numIds=1304000D0AABBBB&denomIds=13.4&selectedTab=summary)  [Betamethasone dipropionate 0.05% scalp lotion : 98.000000000](https://openprescribing.net/analyse/#org=practice&orgIds=15N&numIds=1304000D0AABCBC&denomIds=13.4&selectedTab=summary)  [Betamethasone diprop 0.05%/Salicylic acid 2% scalp applic : 107.000000000](https://openprescribing.net/analyse/#org=practice&orgIds=15N&numIds=1304000D0AABDBD&denomIds=13.4&selectedTab=summary)  [Betamethasone dipropionate 0.064% / Clotrimazole 1% cream : 272.000000000](https://openprescribing.net/analyse/#org=practice&orgIds=15N&numIds=1304000D0AACACA&denomIds=13.4&selectedTab=summary)  [Diprosone 0.05% cream : 12.000000000](https://openprescribing.net/analyse/#org=practice&orgIds=15N&numIds=1304000D0BBAAAA&denomIds=13.4&selectedTab=summary)  [Diprosone 0.05% ointment : 10.000000000](https://openprescribing.net/analyse/#org=practice&orgIds=15N&numIds=1304000D0BBACBA&denomIds=13.4&selectedTab=summary)  [Diprosone 0.05% lotion : 3.000000000](https://openprescribing.net/analyse/#org=practice&orgIds=15N&numIds=1304000D0BBAEBC&denomIds=13.4&selectedTab=summary)  [Diprosalic 0.05%/3% ointment : 365.000000000](https://openprescribing.net/analyse/#org=practice&orgIds=15N&numIds=1304000D0BCAABB&denomIds=13.4&selectedTab=summary)  [Diprosalic 0.05%/2% scalp application : 329.000000000](https://openprescribing.net/analyse/#org=practice&orgIds=15N&numIds=1304000D0BCABBD&denomIds=13.4&selectedTab=summary)  [Lotriderm cream : 276.000000000](https://openprescribing.net/analyse/#org=practice&orgIds=15N&numIds=1304000D0BDABCA&denomIds=13.4&selectedTab=summary) | | | | 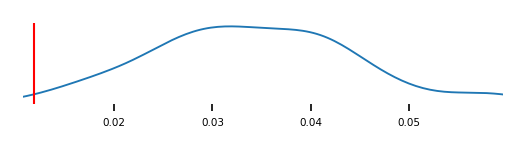 | | | |
| [Fusidic acid](https://openprescribing.net/analyse/#org=practice&orgIds=15N&numIds=1103010H0&denomIds=11.3.1&selectedTab=summary) | 359 | Antibacterials | 13,283 | 0.03 | 0.08 | 0.03 | -2.12 |
| [Fusidic acid 1% modified-release eye drops : 359.000000000](https://openprescribing.net/analyse/#org=practice&orgIds=15N&numIds=1103010H0AAAAAA&denomIds=11.3.1&selectedTab=summary) | | | | 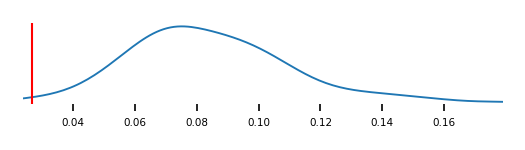 | | | |
| [Senna](https://openprescribing.net/analyse/#org=practice&orgIds=15N&numIds=0106020M0&denomIds=1.6.2&selectedTab=summary) | 39,769 | Stimulant laxatives | 110,838 | 0.36 | 0.55 | 0.10 | -2.03 |
| [Senna 7.5mg tablets : 36954.000000000](https://openprescribing.net/analyse/#org=practice&orgIds=15N&numIds=0106020M0AAACAC&denomIds=1.6.2&selectedTab=summary)  [Senna fruit 12.4% / Ispaghula 54.2% granules : 12.000000000](https://openprescribing.net/analyse/#org=practice&orgIds=15N&numIds=0106020M0AAAJAJ&denomIds=1.6.2&selectedTab=summary)  [Senna 15mg tablets : 605.000000000](https://openprescribing.net/analyse/#org=practice&orgIds=15N&numIds=0106020M0AAAPAP&denomIds=1.6.2&selectedTab=summary)  [Senna 7.5mg/5ml oral solution sugar free : 2117.000000000](https://openprescribing.net/analyse/#org=practice&orgIds=15N&numIds=0106020M0AAATAT&denomIds=1.6.2&selectedTab=summary)  [Senokot 7.5mg tablets : 1.000000000](https://openprescribing.net/analyse/#org=practice&orgIds=15N&numIds=0106020M0BBAEAC&denomIds=1.6.2&selectedTab=summary)  [Senokot Max Strength tablet 12 Years Plus : 29.000000000](https://openprescribing.net/analyse/#org=practice&orgIds=15N&numIds=0106020M0BBAFAP&denomIds=1.6.2&selectedTab=summary)  [Senokot 7.5mg/5ml syrup sugar free : 1.000000000](https://openprescribing.net/analyse/#org=practice&orgIds=15N&numIds=0106020M0BBAGAT&denomIds=1.6.2&selectedTab=summary)  [Senokot 7.5mg tablets Adult : 1.000000000](https://openprescribing.net/analyse/#org=practice&orgIds=15N&numIds=0106020M0BBAJAC&denomIds=1.6.2&selectedTab=summary)  [Manevac granules : 49.000000000](https://openprescribing.net/analyse/#org=practice&orgIds=15N&numIds=0106020M0BFAAAJ&denomIds=1.6.2&selectedTab=summary) | | | | 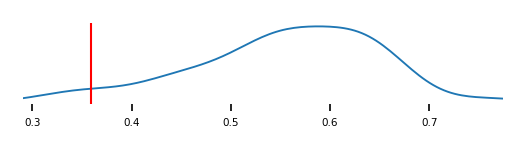 | | | |
| [Ticagrelor](https://openprescribing.net/analyse/#org=practice&orgIds=15N&numIds=0209000Z0&denomIds=2.9&selectedTab=summary) | 2,285 | Antiplatelet drugs | 467,104 | 0.00 | 0.02 | 0.01 | -2.02 |
| [Ticagrelor 90mg tablets : 2148.000000000](https://openprescribing.net/analyse/#org=practice&orgIds=15N&numIds=0209000Z0AAAAAA&denomIds=2.9&selectedTab=summary)  [Ticagrelor 60mg tablets : 97.000000000](https://openprescribing.net/analyse/#org=practice&orgIds=15N&numIds=0209000Z0AAABAB&denomIds=2.9&selectedTab=summary)  [Ticagrelor 90mg orodispersible tablets sugar free : 40.000000000](https://openprescribing.net/analyse/#org=practice&orgIds=15N&numIds=0209000Z0AAACAC&denomIds=2.9&selectedTab=summary) | | | | 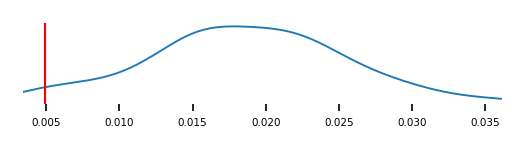 | | | |
| [Lactulose](https://openprescribing.net/analyse/#org=practice&orgIds=15N&numIds=0106040G0&denomIds=1.6.4&selectedTab=summary) | 19,621 | Osmotic laxatives | 127,773 | 0.15 | 0.28 | 0.06 | -1.89 |
| [Lactulose 3.1-3.7g/5ml oral solution : 19424.000000000](https://openprescribing.net/analyse/#org=practice&orgIds=15N&numIds=0106040G0AAAAAA&denomIds=1.6.4&selectedTab=summary)  [Lactulose 10g/15ml oral solution 15ml sachets sugar free : 197.000000000](https://openprescribing.net/analyse/#org=practice&orgIds=15N&numIds=0106040G0AAAGAG&denomIds=1.6.4&selectedTab=summary) | | | | 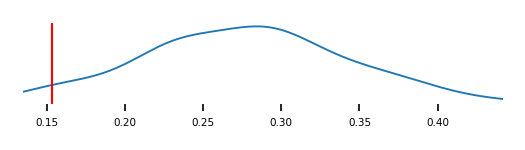 | | | |
