# Supplementary material for: Data-Driven Identification of Unusual Prescribing Behavior: Analysis and Use of an Interactive Data Tool Using 6 Months of Primary Care Data From 6500 Practices in England
Source: JMIR Med Inform. 2023 Apr 19;11:e44237. doi: 10.2196/44237 (PMC10162592; doi:10.2196/44237)

**Multimedia Appendix 2**

**Prototype dashboard showing the top and bottom five outlying chemicals for Cumbria and northeast STP.** BNF Chemical is the chemical of interest, Chemical Items provides the number of prescribing items containing this chemical. BNF Subparagraph is the BNF Subparagraph to which the Chemical belong and Subparagraph Items is the number of prescribing items containing an item belonging to this BNF Subparagraph. Ratio, Mean, std, and Z_Score place the chemical items count in the context of the subparagraph items count as described in the methods. The sparkline plot shows where the Ratio value for this STP occurs (vertical red line) in the context of the same Ratio in all STPs (summarised by the blue line).


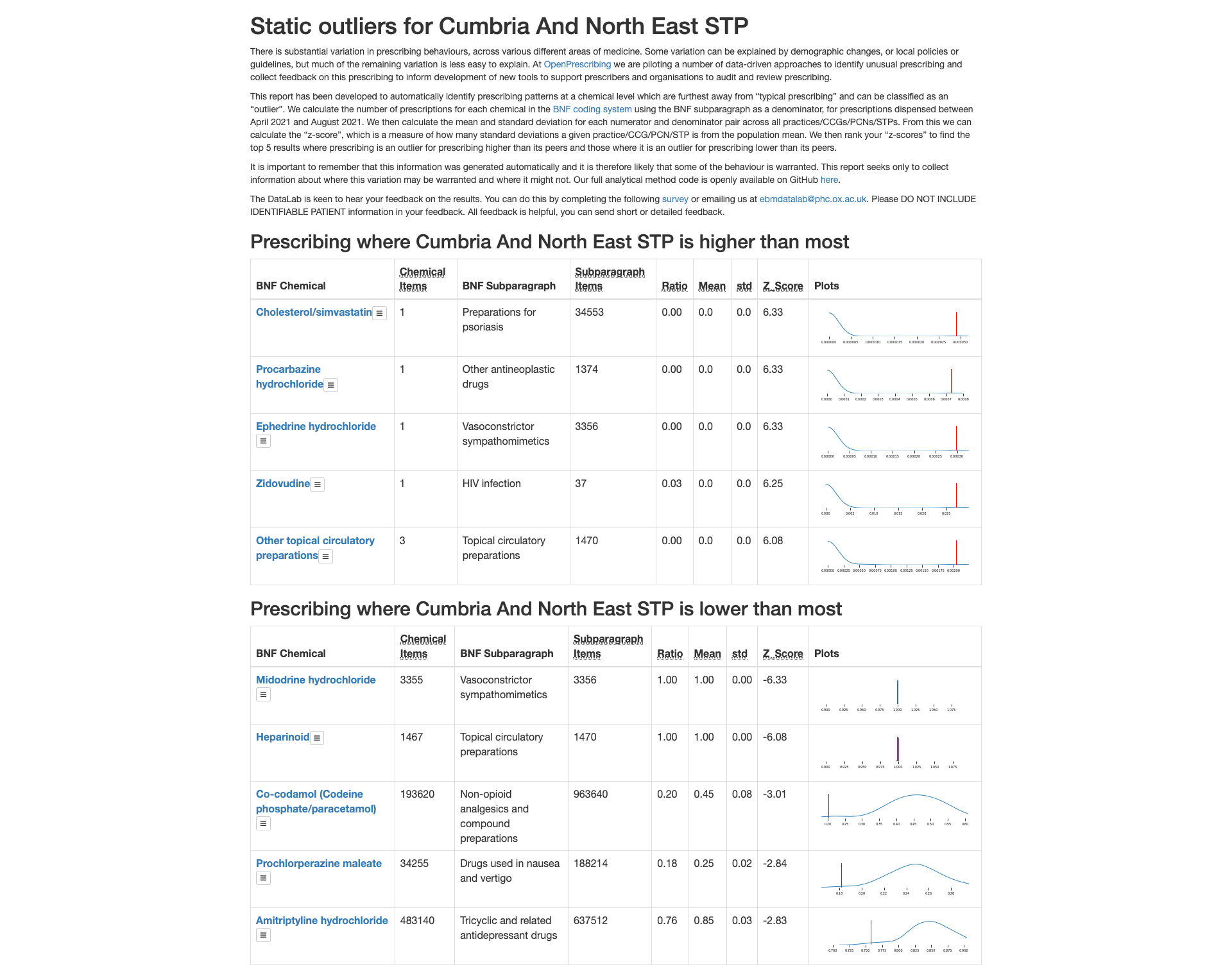

Supplement: Multimedia Appendix 2 [file medinform_v11i1e44237_app2.docx]
